# Diabetic foot ulcer photography study: a study within a trial to assess the reliability of two-dimensional (2D) photography for the assessment of ulcer healing in patients with diabetes-related foot ulcers—protocol paper

**DOI:** 10.1136/bmjopen-2024-090299

**Published:** 2025-01-09

**Authors:** Sarah Tess Brown, Howard Collier, Lucy Askew, Rachael Mary Gilberts, Linda D Sharples, Jane Nixon, Elizabeth McGinnis, Michael Backhouse, Frances Game, Phil Faulks, Myka Ransom, Colin Charles Everett, E Andrea Nelson, David Russell

**Affiliations:** 1 Clinical Trials Research Unit, Leeds Institute of Clinical Trials Research, University of Leeds, Leeds, UK; 2 Medical Statistics, London School of Hygiene and Tropical Medicine, London, UK; 3 Health Sciences, University of Leeds, Leeds, UK; 4 University of Warwick, Coventry, UK; 5 Department of Diabetes, National Health Service, Derby, UK; 6 Digital education Service, University of Leeds, Leeds, UK; 7 Glasgow Caledonian University, Glasgow, UK; 8 Leeds Teaching Hospitals NHS Trust, Leeds, Diabetes Limb Salvage Service, UK

**Keywords:** Diabetic foot, STATISTICS & RESEARCH METHODS, Clinical Trial

## Abstract

**Background:**

The primary endpoint in diabetes-related foot ulcer (DFU) trials is often time to healing, defined as complete re-epithelialisation with absence of drainage, requiring clinical expert assessment as the gold standard. Central blinded photograph review for confirmation of healing is increasingly being undertaken for internal validity. The Diabetic Foot Ulcer Photography study aims to determine the agreement between blinded independent review panel members for assessing ulcer healing status in patients with DFUs.

**Methods and analysis:**

Photographs of ulcers clinically assessed as healed or not healed across 300 participants recruited to one of two randomised controlled trials (MIDFUT and CODIFI2), will be independently reviewed by a central blinded panel consisting of four clinicians with expertise in ulcer healing assessment. Staff at recruiting sites will take photographs using a standardised camera and protocol. Photographs will be reviewed at three levels of magnification: raw image, image standardised to a measurement scale included in the photograph and standardised image with magnification permitted. Reviewers will assess the healing status and their confidence level in making a healing judgement, with reasons reported for a low confidence rating. Analysis at each level of magnification will estimate inter- and intra-rater reliability on the assessments of healing of photographs with the clinical assessment (primary) and confidence rating using multivariable logistic mixed models. Analysis of the learning curve for the assessment of healing and confidence rating will use exponential and two-phase models.

**Ethics and dissemination:**

Ethics approval has been granted by the National Research Ethics Service Committees (MIDFUT 17/YH/0055; CODIFI2 18-WS-0235). All participants will provide a written informed consent for photography before recruited onto the respective study. Photographs will be transferred to the trials’ coordinating centre via a secure file transfer service and saved in a restricted access folder on a secure server. Results will be disseminated via publications in scientific journals and conference presentations.

**Trial registration number:**

MIDFUT (ISRCTN64926597) and CODIFI2 (ISRCTN74929588).

**STRENGTHS AND LIMITATIONS OF THIS STUDY**

- The study design will provide a robust assessment of inter- and intra-rater reliability of diabetes-related foot ulcer two-dimensional (2D) photography in making a healing judgement.
- Photography review will be conducted at three levels of magnification
- The design allows the learning curve for the assessment of healing and confidence rating to be characterised.
- Potential challenges with obtaining a 2D photograph of sufficient quality for review, including limitations due to the curvature and positioning of the ulcer on the foot will be determined.
- Generalisability of results will be limited to chronic DFU.

## Introduction

 The Diabetic Foot Ulcer Photography study was designed to inform future clinical trials and research on whether blinded central review of photographs is reliable for assessing ulcer healing status in participants with DFUs. The study is embedded within two randomised controlled trials (RCTs) in patients with diabetes-related foot ulcers (DFUs), Multiple Interventions for Diabetic Foot Ulcer Treatment Trial (MIDFUT) and CODIFI2. MIDFUT^1^ is a seamless phase II/III, multiarm multistage RCT evaluating, in phase II, the short-term efficacy of the three treatment strategies: hydrosurgical debridement alone or with decellularised dermis or a combination of the two with negative pressure wound therapy (NPWT), as an adjunct to treatment as usual (TAU) compared with TAU. In phase III, the trial aims to compare the clinical and cost-effectiveness of the most promising treatment strategy compared with TAU in the treatment of hard to heal DFUs. CODIFI2 is a phase III trial which aims to evaluate the clinical and cost-effectiveness of tissue and swab sampling techniques, both processed using microbiological standard culture and sensitivity methods, in patients with clinically infected DFUs. In both trials, the primary endpoint is time to healing of the index ulcer, with healing defined as complete re-epithelialisation of the wound surface in the absence of drainage^2^ via blinded outcome assessment of photographs. Additional reasons for taking photographs in MIDFUT/CODIFI2 included verification of the index ulcer location (photograph of the foot) and re-ulceration and to provide a back-up for ulcer area measurement when acetate tracing was not available. Further reasons included for training opportunities, for example, for staff at recruiting sites, to support adequate ulcer debridement prior to ulcer healing assessments.

Expert clinical assessment is considered to be the current gold standard procedure for the assessment of ulcer healing.^3^ To ensure internal validity of trial results, the assessment of ulcer healing should be conducted by an independent assessor who is blind to treatment group allocation. In trials assessing time to healing, there is a potential risk of bias, in part, due to the preference for a time to event outcome, rather than the proportion of ulcers that are healed at a prespecified time point, for example, at 12 or 52 weeks, the latter approach being simpler to report and analyse. Moreover, a less refined judgement may be applied to decision-making on the healing outcome at a specified time point, whereas in a trial with time to event outcome, there may be a fine judgement between ‘almost healed’ and ‘just healed’. In DFU trials, healing assessment is sometimes supplemented by a further review undertaken via a blinded central review panel, consisting of clinicians with expert knowledge to assess healing. However, provision of only photographs of ulcers clinically assessed as healed could risk over-reporting of healing. It is therefore important to mitigate this risk by also providing a set of photographs of ulcers that have previously been assessed clinically as not healed.

Only one study in chronic DFUs using photography for the assessment of healing was identified, suggesting this practice is uncommon in this clinical indication. The study evaluated the efficacy of dehydrated human amnion/chorion membrane allograft in the management of DFUs,^4^ in which all photographs, taken post debridement, were reviewed by three independent clinicians to determine the timing of complete healing, achieved via consensus.

An RCT of DFU with surgical wounds healing by secondary intention (SWHSI-2),^5^ included patients with surgical debridement of a DFU as a subset, comparing NPWT with usual care over 12 months. A standardised photograph was taken of the wound at the first healing assessment. Participants (with assistance from family/friends if necessary) were also asked to take a digital photograph of the wound themselves and submit this to the study team. Study specific instructions were provided to facilitate this. The photographs were used to facilitate healing verification by clinically experienced, independent, blinded reviewers.

A systematic review reports the existing literature on the available wound assessment and monitoring systems for DFUs.^6^ However, there were no studies reporting the reliability of photographs for the assessment of healing. Hence, the lack of reported studies suggests there is a large evidence gap on the use of photography in chronic DFUs.

The purpose of the photography review process in the Diabetic Foot Ulcer Photography study is two-fold: first, to fulfil the funder’s requirement of having a blinded photography review of the primary endpoint data and, second, to undertake a study to assess the reliability of using photographs as a method of assessing healing. The focus of this paper is on the latter. The specific objectives of the Diabetic Foot Ulcer Photography study are to:

1. Determine the extent of agreement on the assessment of healing status between two-dimensional (2D) photographs, at each of three levels of magnification, with the clinical assessment both between (inter-rater reliability) and within reviewers (intra-rater reliability).
2. Estimate the learning curve for the assessment of healing status and determine the point at which learning is complete for the assessment of healing status.
3. Estimate the learning curve for the confidence rating on the assessment of healing.
4. Explore reasons for low confidence rating.

In clinical practice, photographs of ulcers, or site of the healed ulcer, are typically taken using a non-standardised approach, resulting in large variation in the quality of the images. Even with a standardised protocol, including camera set-up, lighting conditions and distance from the ulcer etc, it is anticipated there will be potential variation in the quality of photographs taken in ambient light conditions and level of magnification of the images. To compensate for this variation, three levels of review are planned in this study. The first level corresponds to reviewing the ‘raw’ image (without magnification), as taken by a member of the research team, thereby more closely reflecting the variation observed in clinical practice. The second level involves cropping the image to the measurement scale placed adjacent to the ulcer, or site of the healed ulcer, to standardise the size of the image, thereby removing the variation in distance between the ulcer and camera. For the third level of review, magnification of the standardised images is permitted to enable reviewers to make a clearer judgement on the healing status; the level of magnification at which a decision on healing status is made is recorded.

To explore practical issues with making a healing judgement, reviewers will provide a confidence rating in their judgement of healing status, with reasons reported for images with a low confidence rating, including poor lighting, blurred picture, shape or position of ulcer. Exploring these issues will help with informing future guidance on standardisation of the photographic technique at sites.

The learning curve for assessment of healing is expected to be minimal. However, as the existing evidence is limited to clinical opinion, the study provides the opportunity for a formal assessment. The probability of agreement between photography review and clinical assessment will be modelled over time for each panel member assuming two phases (a learning phase and a postlearning phase).^7^ The point where these two phases meet will provide a recommendation for the number of reviews before results are consistent, that is, the learning phase is complete.

## Methods and analysis

**Figure 1:**
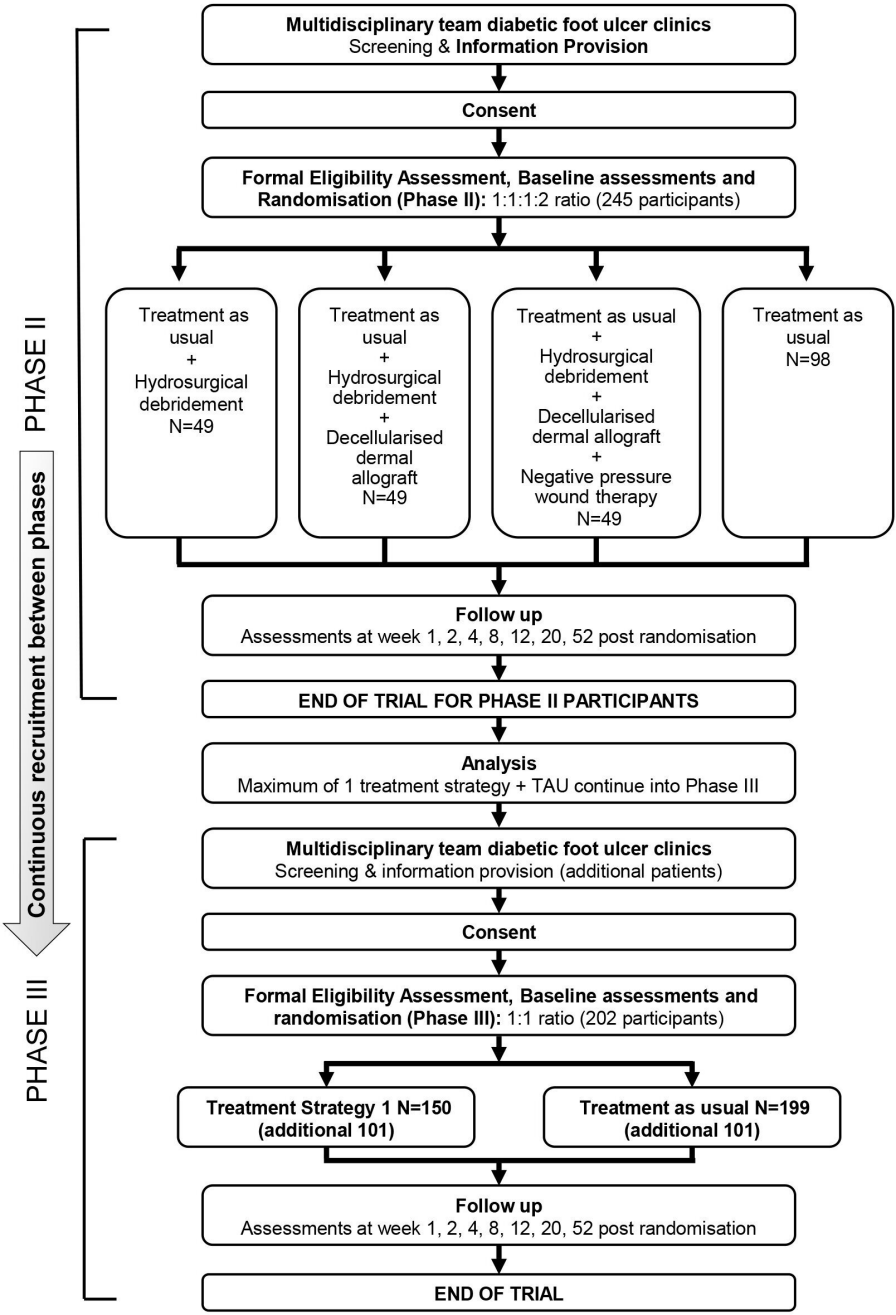
MIDFUT flow diagram. MIDFUT, Multiple Interventions for Diabetic Foot Ulcer Treatment Trial; TAU, treatment as usual.

The flow diagrams for MIDFUT and CODIFI2 RCTs are presented in figures1  2, respectively.

**Figure 2:**
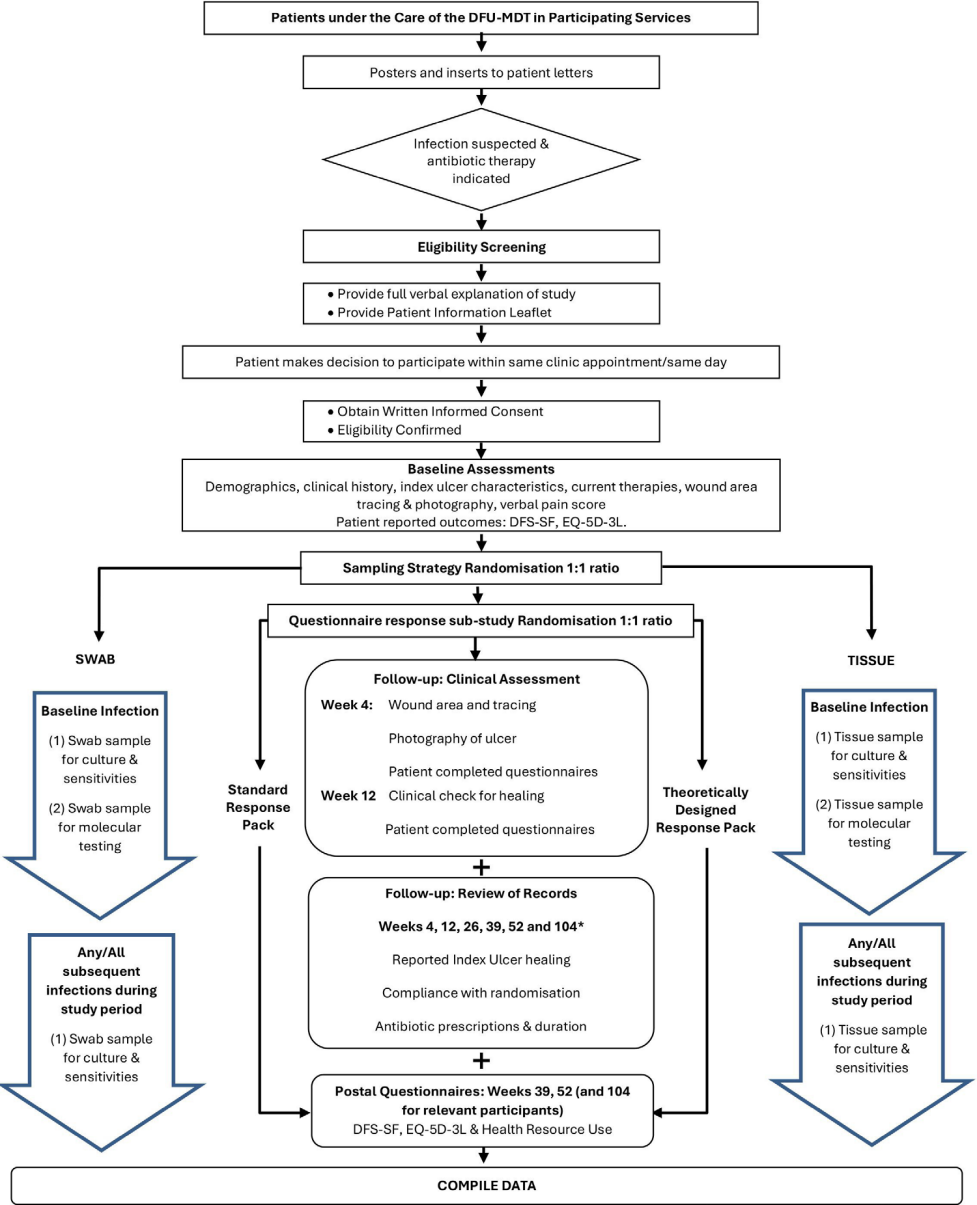
CODIFI2 flow diagram. DFU, diabetes-related foot ulcer.

### Consent and data collection process

In both RCTs, photography is used to establish the status of the index ulcer and is compulsory for all participants at the time of entering the study. Verbal agreement will be confirmed before each photograph is taken, and the participant can refuse to be photographed at any time yet remain in the study. Wording in the consent forms allows for sharing of photographs in the context of this study.

In both RCTs, photographs of the foot to establish the location of the index ulcer and of the index ulcer itself will be taken at the baseline visit.

In MIDFUT, follow-up photographs will be taken of the index ulcer at week 2, and by an assessor blinded to treatment allocation at week 4, at the initial visit and confirmation of healing visit. Note that participants require maintenance of healing for 2 weeks to be classed as healed. A further photograph of the foot will be taken if re-ulceration of the index ulcer is reported to confirm the site of re-ulceration by the blinded assessor. A 25% random sample of all participants will be selected at randomisation, for a photograph of the index ulcer to be taken if unhealed at weeks 12, 20 and 52 visits, to provide a subset for unhealed ulcer photographs for review. Note that the photograph of the index ulcers in MIDFUT will be taken following sharp, non-surgical debridement to remove callous and non-viable tissue, if clinically indicated.

In CODIFI2, follow-up photographs of the index ulcer by a blinded assessor will be taken at week 4 and at the confirmation of healing visit. Note that, in this study, confirmation of healing is conducted at a single assessment. A 50% random sample of the first two participants recruited at each site, followed by a 25% random sample of all participants thereafter, will be selected at randomisation to have a photograph of the index ulcer if unhealed at the week 26 visit as a record of healing progress at that point (should further data not be available for any reason) and to provide for a bank of unhealed photos for the photography study.

A subset of photographs for evaluation in the Diabetic Foot Ulcer Photography study will be selected across the following visits:

- MIDFUT and CODIFI2: healing visits.
- MIDFUT: subset of participants with unhealed index ulcers at baseline and weeks 12, 20 and 52.
- CODIFI2: subset of participants with unhealed index ulcers at baseline and week 26.

The selected study camera (camera model ‘Canon IXUS 185’) has been supplied to sites together with a protocol detailing the use of a standardised photographic method including the use of a measurement scale with ruler. The camera will be used on the ‘automatic’ function, corresponding to the default setting. For consistency, future processing of the images and interpretation, it is important that only the study camera provided to sites is used to take photographs. The protocol provides clear instructions on the anonymisation, secure transfer to the trials’ coordinating centre (Leeds Institute of Clinical Trials Research (LICTR)) and deletion of the photographs to ensure standardisation across all sites. LICTR will log each photograph and assign a unique 6-digit number to anonymise the photograph prior to review.

### Process for standardising the photographs

LICTR will process raw images using Adobe Lightroom Classic CC.^8^ To white balance the image, a known white colour included in a trial grey scale card will be selected using the ‘White balance selector’. ‘Lens Correction’ will be enabled by selecting the ‘Remove Chromatic Aberration’ and ‘Enable Profile Corrections’ options. The correct camera make in the lens profile section will also be selected. To correct the verticals in the image, the ‘Global Upright Tool’ will be used to draw straight lines using the scale as a reference. For optimum results, two horizontal and two vertical lines are positioned using the scale creating a square around the image. The Crop Overlay tool will be used to change the aspect ratio to 1:1 (1×1) for a square image crop. The image is positioned by cropping out any identifiers (participant initials, date of birth, visit number, Trial ID) and ensuring the whole image is within the grid. Once in position, the Crop Overlay tool is re-selected to display the processed image. The images will then be saved using the recommended settings (image format, JPEG; colour space, sRGB; quality, 100%). To ensure all files are the same size, Image Sizing will be used by selecting ‘Resize to fit’ and clicking ‘Width & Height’. Width will be changed to 1500 pixels and Height to 1500 pixels. Resolution is set to 300 pixels per inch. To remove all identifiable metadata, ‘Copyright only’ will be selected. Following processing, images will be saved in the relevant folder for review. All images will then be deleted from Adobe Lightroom.

A set of test photographs of unhealed ulcers from participants not selected for the photography study in MIDFUT and a further set of photographs of healed DFUs provided by one of the reviewers (from a separate trial) have been used in preliminary analysis for developing the process and protocol. A medical photographer developed the process and produced a video animation for incorporating into the work instruction.

### Sampling of photographs

Photographs, with a predefined ratio of 1:1 for healed/non-healed ulcers, will be selected from the full set of photographs across the study assessment timepoints. For the assessment of intra-rater reliability, four reviewers will receive a randomly selected subset of 30 previously reviewed photographs by the panel of reviewers using the same pre-defined ratio (see section ‘Review of photographs’).

### Review of photographs

The central blinded panel of reviewers will comprise four clinical members (diabetologist, tissue viability nurse, podiatrist, vascular surgeon) of the trial management groups (TMGs) of MIDFUT and CODIFI2 who will not be aware of each participant’s identity, trial, treatment group or time point at which the photograph was taken. The decision for having four reviewers is based on ensuring the main clinical disciplines are covered and having the requisite experience and skills to make an assessment of healing and their availability within the trial teams. A protocol detailing the process for reviewing the photographs together with the requirements relating to confidentiality, storage and subsequent destruction of the photographs will be signed off by all reviewers, prior to starting the photography review process. The four clinical members of the trial TMGs will independently review all photographs.

Photographs of unhealed ulcers and healed ulcers will be assessed in the same batch to maintain the blind and thereby reduce the risk of overreporting of healing.

Photographs will be reviewed at the following three levels of magnification:

- Raw: unmagnified photographs.
- Standardised: photographs cropped to a standard size using the measurement scale in the photograph, as described above.
- Standardised with magnification permitted: magnification of the standardised photographs in (ii). The extent of magnification required to make a judgement on healing status is controlled by the reviewer.

The assessment of healing at magnification levels (i) and (ii) will be conducted by all four reviewers. Review of (iii) will take place by three reviewers with access to the software Paint.net,^9^ freely available for Microsoft Windows. The review of photographs for each level of magnification will be conducted on different occasions.

The monitor screen size (minimum of 17 inch) used will be recorded for each reviewer. Review of the images where magnification is permitted will take place using the software Paint.net. Reviewers outside University of Leeds will use a work laptop/personal computer (PC) (University/NHS) which is encrypted and secure. Note that reviewers internal to LICTR will have direct access to the secure network. Photographs will be saved to the laptop/PC temporarily for the review session and will then be deleted.

Information on the location of the ulcer will be provided to reviewers. No further clinical information is to be provided. Reviewers will enter data directly onto a bespoke MACRO database at the point of reviewing photographs. For each photograph, reviewers will report a confidence level in their assessment of making a healing judgement on an adapted 11-point scale ranging from 0 (not confident at all) to 10 (very confident)^10^. If confidence rating is low (0–3), then the reviewer will record the reason to aid interpretation of results. Reviewers will then enter their assessment of healing status (healed/not healed/unable to assess) onto the database. For reviews in which magnification of the image is permitted, the level of magnification required to make a healing assessment is recorded. A minimum of 6 weeks between photography review sessions is planned based on feasibility while also ensuring low risk of recall bias.

### Sample size

The estimand of interest is inter-rater reliability, measured by kappa, a measure of chance-corrected agreement. A total of 300 photographs from independent participants will provide a minimum precision of±0.10 (corresponding to the half width of the 95% CI) if kappa is k=0.5. The assessment of inter-rater reliability will be conducted on all 300 photographs at each level of magnification. The assessment of intra-rater reliability, whereby each reviewer will conduct a repeat review of a set of 30 previously reviewed photographs, is based on feasibility.

### Statistical analysis

**Figure 3:**
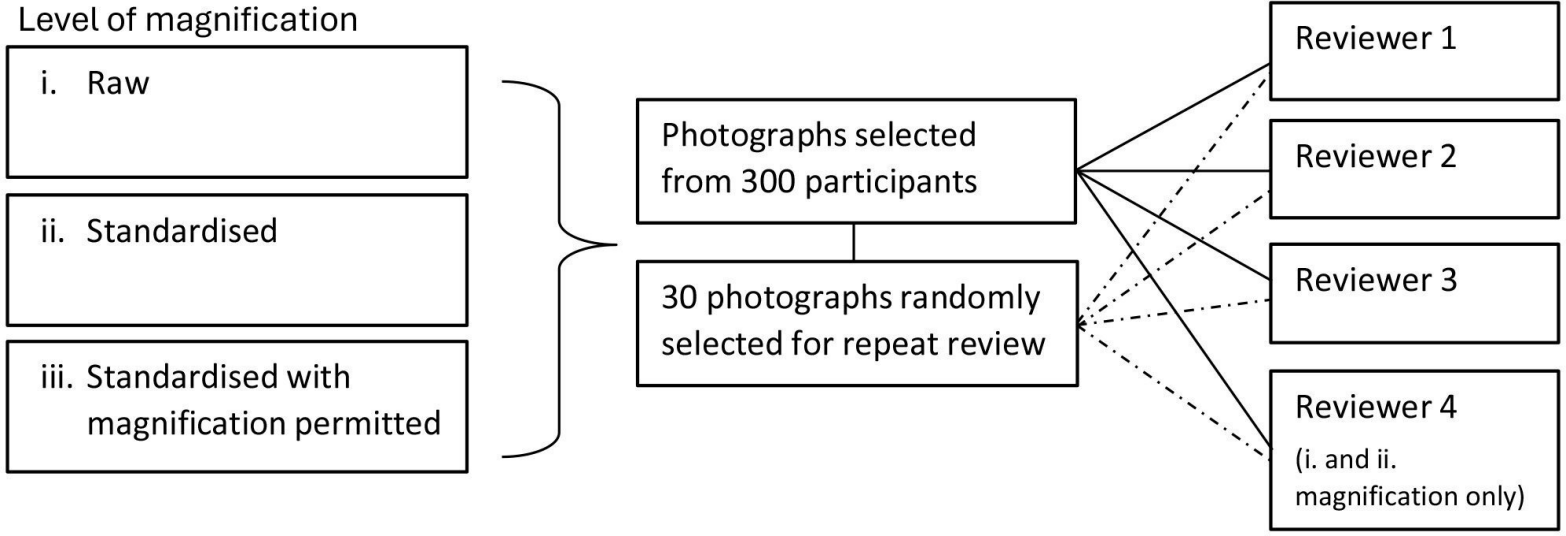
Number of photographs at each level of magnification.

The full set of photographs selected from a total of 300 participants will be included in the analysis for each level of magnification (see figure 3). The data for each level of magnification will be modelled separately.

### Healing assessment

#### Primary endpoint analysis

##### Extent of agreement of healing status with clinical assessment (gold standard), between and within reviewers

Cross-tabulations of healing status between photography and clinical review, by reviewer and repeated reviews, will be presented.

### Primary analysis

Agreement with the outcome is coded as: 1 if both clinician and photography are assessed as healed or if both are assessed as not healed and 0 if either clinician assessment is healed and photography assessment is not healed or vice versa. Data will have a hierarchical structure (see online supplemental figure S1). A multivariable logistic mixed model will be fitted on the response, ‘agreement with clinical assessment’ with covariates, healed status (yes/no), trial (MIDFUT, CODIFI2), anatomical site (forefoot, mid/hindfoot) and presentation of the index ulcer (DFU, surgical debridement wound, open minor amputation); participant and reviewer will be included as crossed random effects. Adjusted odds ratios (ORs) relative to the average odds of agreement and corresponding 95% CIs for fixed effects will be reported. Further, adjusted intracluster correlation coefficients (ICCs) and 95% CIs will be reported, along with the components of variation attributable to participants, reviewers and repeated measurements. If any random effects variances are close to zero, alternative simplified hierarchical models will be fitted and reported. The model will be fitted using likelihood-based methods in SAS (V.9.4) and model fit assessed by plotting random effects distributions and comparing model-predicted proportions of agreements against observed proportions. For photographs reviewed twice, Kappa’s coefficient of agreement, estimated using methods of by Fleiss, will be reported.^11^

For the *learning curve analysis,* the probability of agreement between photography review and clinical assessments over time will be modelled using a non-linear function on the logit scale. An exponential and a two-phase model will be fitted for each level of magnification using methods developed by Papachristofi *et al*^7^for each reviewer separately to assess their individual learning curve . Estimates of the model parameters from all reviewers will be combined using methods developed for meta-analysis to obtain a group-average learning curve.

### Secondary endpoint analysis: extent of agreement in confidence rating between and within reviewers

Cross-tabulations of confidence rating for each reviewer and repeated reviews will be presented by healing status and overall. For each level of magnification, a multivariable linear mixed-effects regression model will be fitted to the confidence rating with fixed and random effects as above. Adjusted means, ICCs and corresponding 95% CIs will be reported.

For the *learning curve analysis*, a multivariable linear regression model will be fitted to the confidence rating for each reviewer, with an exponential term for the order of assessment. Models for each reviewer will be meta-analysed to provide a group-average learning curve.

Given the design of the photography study, few missing measurements are expected. Missing measurement patterns will be explored in the study dataset. As the structure of the data is hierarchical, parameter estimates will be unbiased provided that data are ‘missing at random’. Moreover, random effects analysis methods will be employed which respect the structure of the data.^12^

### Study status

The first batch of photographs were sent to the central panel in November 2021. As of 20 October 2024, a total of 61 raw photographs and 49 standardised photographs with magnification permitted have been reviewed. The final review of photographs is expected to take place by end of February 2025.

### Patient and public involvement

Patient and public involvement was central to the design of both trials, actively helping to shape discussions and decisions through written feedback and group discussion. Specific feedback relating the Diabetic Foot Ulcer Photography study included agreement to completion of photographs which may be considered a burden and the request for a plain English summary of the proposed analysis methods.

## Discussion

This Diabetic Foot Ulcer Photography study is the largest planned study in DFUs research to evaluate photographic information on ulcer healing outcome assessment, including a robust assessment of inter- and intra-rater reliability, confidence rating and evaluation of the learning curve in making a healing judgement. It also allows the opportunity to assess the quality of photographs taken for the assessment of healing and to use this information to benchmark and address in future clinical studies and practice.

Results of Diabetic Foot Ulcer Photography study will help to establish whether future studies should use blinded central review of photographs for ulcer healing assessments in patients with DFUs. If demonstrated, blinded photography review could potentially replace the need for an ‘in person’ independent assessment to confirm healing.

The Diabetic Foot Ulcer Photography study uses a standardised approach for taking the photographs, with a secure process for transfer and review. Photographs will be taken using the automatic (default) setting on the camera which may allow for generalisability to photographs taken on later models of 2D cameras. Moreover, assessment of healing using 2D more closely reflects the process of monitoring healing in current clinical practice. However, it is anticipated that having three levels of review in the study will entail a heavy resource and time commitment in the post processing of photographs. Post processing is required at two levels of review: first, to redact participants’ identifiers and anonymise the raw images and, second, to standardise all the selected anonymised raw images by cropping the image to the measurement scale included in the photograph using instructions provided by the medical photographer. The process of standardisation is expected to take approximately 5 min per image or 25 hours for a total of 300 photographs. Limitations for this second stage of processing include the requirement of a measurement scale in the photograph; if research staff at sites do not include this, the photograph cannot be included in this level of review, nor the stage where magnification is permitted. Moreover, the team members involved in post processing of images are not trained medical photographers, and hence the level of accuracy and consistency achieved in this processing may be compromised to some extent.

Recording the reviewers’ levels of confidence in making healing judgments allows for the reasons for low confidence ratings to be explored. This will assist with informing future guidance on taking photographs of DFUs including appropriate preparation prior to the assessment of healing.

The Diabetic Foot Ulcer Photography study provides the opportunity to characterise the learning curve for the assessment of healing and confidence rating. A large difference in confidence ratings between reviewers earlier on is expected to relate to a large difference in confidence in the assessment of healing between reviewers. As the confidence ratings come closer together, the residual difference in confidence is likely to be explained by the quality of the photograph.

Potential limitations of this study include capturing an accurate representation of the healed/unhealed ulcer using a 2D image, given the curvature and positioning of the ulcer on the foot. As a result, there may be challenges with obtaining a photograph of sufficient quality for review. Although three-dimensional (3D) photography methods were considered at the trial design stage, the technology was expensive and not sufficiently reliable at that time. A systematic review^6^ reported a total of 18 commercially available wound assessment and monitoring systems (mobile applications or devices) listed on WoundSource.^13^ The increasing range of 3D and integrated systems for wound assessment allows further choice. However, information on the analytical performance of these systems is limited.

Artificial intelligence (AI) methods for wound assessment and monitoring have also increased within the last decade, including the use of neural networks,^14^ support vector machines^15^ and random field modelling approaches.^16^ Limitations reported for these methods include the need for a larger database of wound images to improve the accuracy of the machine learning model^15 16^ and to increase representation of different ethnicities.^14^ AI methods for determination of wound margins and therefore healing status are still in their infancy. Other less accessible methodology exists, and evaluation studies are ongoing.^17^ Hence, there is still a place for the evaluation of 2D photography for the assessment of healing in clinical research and practice.

Photographs in MIDFUT and CODIFI2 were taken by research staff rather than by the participants, to reflect research and current practice more closely. More recently, there is a move towards allowing patients to take ‘selfies’ of their DFU for clinical review and assessment. A recent pilot study evaluating the use of a smart phone based ‘foot selfie’ system for the early evaluation and monitoring of healing of DFUs was reported.^18^ In SWHSI-2, the trial results of photographs taken by participants to support verification of healing status via a blinded clinical review are still to be published. Hence, the reliability of remote photography for healing assessment is yet to be demonstrated before being implemented in clinical research and practice. Establishing reliability is particularly problematic for DFU due to the wound being on the plantar aspect of the foot in a patient group with restricted joint mobility so that ‘selfies’ are more challenging than wounds in other areas.

## Ethics and dissemination

Ethics approval has been granted by the National Research Ethics Service Committees (REC references: MIDFUT 17/YH/0055; CODIFI2 18-WS-0235). All participants will provide written informed consent for photography before recruited onto the respective study.

Photographs, once taken, will be safely transferred to the trials’ coordinating centre (LICTR) via a secure file transfer service. Once received, photographs will be downloaded and saved in a restricted access folder on the secure server at LICTR. Sites will then delete the photographs from the camera memory. LICTR will log each photograph and assign a unique 6-digit number to anonymise the photograph prior to review.

Results of the Diabetic Foot Ulcer Photography study will be disseminated via publications in scientific journals and conference presentations.

## supplementary material

10.1136/bmjopen-2024-090299 online supplemental file 1
