# Supplementary material for: Diabetic foot ulcer photography study: a study within a trial to assess the reliability of two-dimensional (2D) photography for the assessment of ulcer healing in patients with diabetes-related foot ulcers—protocol paper
Source: BMJ Open. 2025 Jan 9;15(1):e090299. doi: 10.1136/bmjopen-2024-090299 (PMC11752009; doi:10.1136/bmjopen-2024-090299)
Supplement: online supplemental file 1 [file bmjopen-15-1-s001.pdf]

Supplementary file

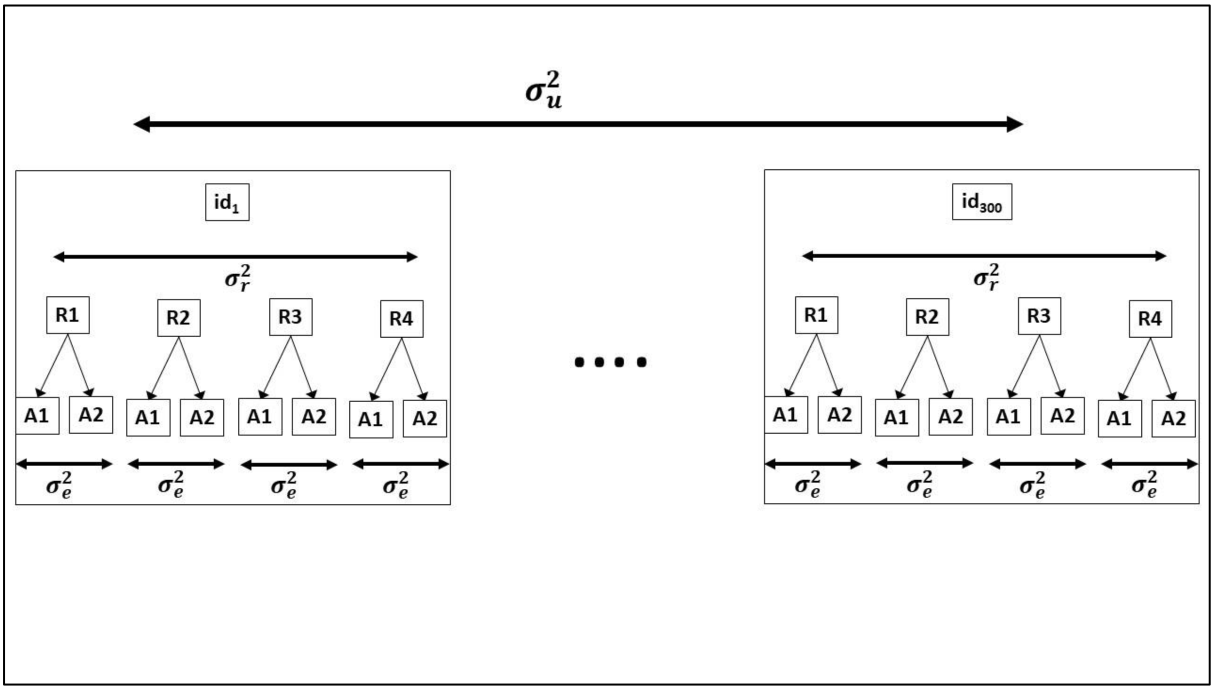

Figure S1: Hierarchical structure of the data

Within each participant we will have four reviewers and their agreement rates may vary. A subset of participants will have their photographs reviewed a second time and there may be variation in agreement rates between these repeated assessments.

This main analysis will estimate the components of total variance in the probability of agreement that are explained by:

- within-reviewer repeated assessments (represented by σ_e^2 in Figure S1),
- between reviewers (represented by σ_r^2),
- between participants (represented by σ_u^2).
