# Supplementary material for: Diabetic foot ulcer photography study: a study within a trial to assess the reliability of two-dimensional (2D) photography for the assessment of ulcer healing in patients with diabetes-related foot ulcers—protocol paper
Source: BMJ Open. 2025 Jan 9;15(1):e090299. doi: 10.1136/bmjopen-2024-090299 (PMC11752009; doi:10.1136/bmjopen-2024-090299)
Supplement: Supplementary file 2 [file bmjopen-2024-090299.reviewer_comments.pdf]

# PEER REVIEW HISTORY

BMJ Open publishes all reviews undertaken for accepted manuscripts. Reviewers are asked to complete a checklist review form and are provided with free text boxes to elaborate on their assessment. These free text comments are reproduced below.

**ARTICLE DETAILS**

**Title (Provisional)**

Diabetic Foot Ulcer Photography Study: Study Within a Trial to assess the reliability of 2D photography for the assessment of ulcer healing in patients with Diabetes-related Foot Ulcers – Protocol Paper

**Authors**

Brown, Sarah Tess; Collier, Howard; Askew, Lucy; Gilberts, Rachael Mary; Sharples, Linda; Nixon, Jane; McGinnis, Elizabeth; Backhouse, Michael; Game, Frances; Faulks, Phil; Ransom, Myka; Everett, Colin Charles; Nelson, E. Andrea; Russell, David

---

**VERSION 1 - REVIEW**

---

| | |
|---|---|
| **Reviewer** | **1** |
| **Name** | **Schwarzer , Saskia** |
| **Affiliation** | **Liverpool Hospital** |
| **Date** | **28-Aug-2024** |
| **COI** | **nil** |

---

Very clear manuscript clearly addressing the aims outlined.

Well written, no suggestions or recommendations.

---

| | |
|---|---|
| **Reviewer** | **2** |
| **Name** | **Basiri, Reza** |
| **Affiliation** | **University of Toronto** |
| **Date** | **01-Sep-2024** |
| **COI** | **None** |

---

Review of the manuscript titled "Diabetic Foot Ulcer Photography Study – Protocol Paper"
**Summary:**
The study intends to evaluate photographic information on ulcer healing outcome assessment, including a robust assessment of inter- and intra-rater reliability, confidence rating and evaluation of the learning curve in making a healing judgment. The study's outcome will help to establish whether future studies should utilize a blinded central review

of photographs for ulcer healing assessments in patients with DFUs, as well as to characterize the learning curve for the assessment of healing.

**Grammatical, Formatting, And Word Choice Comments:**

1. On page 1, “Diabetic Foot Ulcer photography” if this is a name, then all words need to be capitalized; if not, the DFU abbreviation should be used.
2. Please cite the terms at the first appearances (e.g MIDFUT)
3. Figures 2 and 3 have missing text due to small size boxes.
4. Ensure consistent use of terms. For instance, if "blinded reviewers" is used in one section, it should be used consistently instead of switching to "review panel" or "assessors" without clear context.

**Content Comments:**

1. The protocol does not fully explain how certain key values were determined, such as the decision to use four reviewers (page 8); why those specific four medical experts? How does their expertise influence the visual inspection of DFU images? Why “30” previously reviewed photographs (page 8), and why the minimum “six-week” gap between review sessions? While these choices might be based on practical considerations or existing standards, the lack of explicit justification raises questions about whether these values were optimally chosen.
2. This labor-intensive process raises concerns about the scalability and practicality of implementing this approach in broader clinical practice. What is the motivation to substitute one in-person blinded assessor with four virtual blinded assessors? Are there cost-saving benefits? Please elaborate.
3. The protocol briefly mentions considering 3D imaging and AI but ultimately focuses on 2D photography due to concerns about cost and reliability. However, given the rapid advancements in AI and imaging technologies, the study might miss an opportunity to explore more cutting-edge methods that could offer superior accuracy and reproducibility in wound assessment. There have been many advancements in automated DFU detection and classification. Has the author examined those to rationalize when conducting this study? The AI studies would still require remote image annotators, so perhaps the author could discuss how evaluating the remote assessors goes hand in hand with the AI-related annotation task.
4. The title is misleading or perhaps should be narrowed down. While “photography” is included, this protocol only focuses on 2D RGB images. There are many different kinds of photography, such as thermal imaging and depth imaging (not necessarily 3D imaging), which are more reliable and relevant to DFU. Perhaps something more

specific, similar to “Evaluation of 2D RGB Imaging for Diabetic Foot Ulcer Healing Assessment,” would be better.

5. Missing data is an inevitable part of any clinical trial. This protocol should explain how the study will handle missing data. The potential consequences of not addressing this issue include bias in the results or reduced statistical power.
6. In support of number 3 and 4 comments, the authors should examine other relevant DFU data collection protocols such as (Basiri, R., Manji, K., LeLievre, P.M. et al. Protocol for metadata and image collection at diabetic foot ulcer clinics: enabling research in wound analytics and deep learning. BioMed Eng OnLine 23, 12 (2024). https://doi.org/10.1186/s12938-024-01210-6), where a multimodal and comprehensive DFU data collection was conducted. While 2D imaging is the most accessible and feasible approach in DFU image collection, they significantly lack the essential contextual information required for healing assessment. Exploring or adding
temperature factors (thermal imaging or just simple temperature values) may strengthen the scalability and practicality of the protocol, as this parameter is the most crucial element missing from the 2D DFU images.

---

## VERSION 1 - AUTHOR RESPONSE

**Review 1**

We thank Dr. Saskia Schwarzer for her review and consideration of the manuscript.

**Review 2**

We thank Dr. Reza Basiri for his review and consideration of the manuscript.

**Grammatical, Formatting, And Word Choice Comments:**

1. On page 1, “Diabetic Foot Ulcer photography” if this is a name, then all words need to be capitalized; if not, the DFU abbreviation should be used. All words have now been capitalised in the revised manuscript.

2. Please cite the terms at the first appearances (e.g MIDFUT). The trial acronym MIDFUT has been stated in full at the first appearance. However, CODIFI2 is not an acronym for the trial so no update has been here.

3. Figures 2 and 3 have missing text due to small size boxes. Figures 2 and 3 have been updated.

4. Ensure consistent use of terms. For instance, if "blinded reviewers" is used in one section, it should be used consistently instead of switching to "review panel" or "assessors" without clear context.

To clarify, the phrase “blinded assessor” corresponds to the person in clinic making an in-person assessment of healing status, while “blinded reviewers” corresponds to the members of the central review panel who will review the photographs. The manuscript has been updated where appropriate.

**Content Comments:**

1. The protocol does not fully explain how certain key values were determined, such as the decision to use four reviewers (page 8); why those specific four medical experts? How does their expertise influence the visual inspection of DFU images? Why “30” previously reviewed photographs (page 8), and why the minimum “six-week” gap between review sessions? While these choices might be based on practical considerations or existing standards, the lack of explicit justification raises questions about whether these values were optimally chosen.

The choice of the specific four clinical experts for the review panel is based on ensuring the main clinical disciplines are covered and having the requisite experience and skills to make an assessment of healing, and their availability within the trial teams. This rationale is now included in the manuscript.

The assessment of intra-rater reliability, whereby each reviewer will conduct a repeat review of a set of 30 previously reviewed photographs, is based on feasibility as already stated in the manuscript.

A minimum of six weeks between photography review sessions is planned based on feasibility while also ensuring low risk of recall bias. This is now incorporated in manuscript.

2. This labor-intensive process raises concerns about the scalability and practicality of implementing this approach in broader clinical practice. What is the motivation to substitute one in-person blinded assessor with four virtual blinded assessors? Are there cost-saving benefits? Please elaborate.

Confirmation of a healing event by an in person blinded assessor is required for the primary endpoint analysis in both MIDFUT and CODIFI2 trials. The further blinded assessment undertaken via review of photographs (via a central blinded review panel) is an additional requirement set by the funder of the trials. We do not suggest it is implemented in clinical practice. However, we have taken the opportunity to add value to both trials by developing this photography study, the design of which should allow us to address the research question on whether review of 2D photographs is a reliable method for assessing ulcer healing status in patients with DFUs. Moreover, with four reviewers in the central blinded review panel, we can obtain an estimate of between-assessor variability, which is not possible with one person.

3. The protocol briefly mentions considering 3D imaging and AI but ultimately focuses on 2D photography due to concerns about cost and reliability. However, given the rapid advancements in AI and imaging technologies, the study might miss an opportunity to explore more cutting-edge methods that could offer superior accuracy and reproducibility in wound assessment. There have been many advancements in automated DFU detection and classification. Has the author examined those to rationalize when conducting this study? The AI studies would still require remote image annotators, so perhaps the author could discuss how evaluating the remote assessors goes hand in hand with the AI-related annotation task.

Examining more advanced methods for making a healing assessment are outside the scope of this study and would be too costly, primarily because we do not have access to the technology suggested within the "parent" MIDFUT and CODIFI2 trials. Development of methods and expertise would be required which cannot feasibly be conducted for this study alone. An evaluation of 2D photography for assessing healing remains useful and informative for DFU clinics and researchers not using 3D imaging or AI. In addition, Funders of trials still mandate 2D photography is undertaken for a central blinded assessment of healing; this study will inform Funder decision making in the future.

4. The title is misleading or perhaps should be narrowed down. While "photography" is included, this protocol only focuses on 2D RGB images. There are many different kinds of photography, such as thermal imaging and depth imaging (not necessarily 3D imaging), which are more reliable and relevant to DFU. Perhaps something more specific, similar to "Evaluation of 2D RGB Imaging for Diabetic Foot Ulcer Healing Assessment," would be better.

The title has been updated to clarify this is 2D photography.

5. Missing data is an inevitable part of any clinical trial. This protocol should explain how the study will handle missing data. The potential consequences of not addressing this issue include bias in the results or reduced statistical power.

The following paragraph is now included in the manuscript.

Given the design of the photography study, few missing measurements are expected. Missing measurement patterns will be explored in the study dataset. As the structure of the data is hierarchical, parameter estimates will be unbiased provided that data are "missing at random". Moreover, random effects analysis methods will be employed which respect the structure of the data (1).

6. In support of number 3 and 4 comments, the authors should examine other relevant DFU data collection protocols such as (Basiri, R., Manji, K., LeLievre, P.M. et al. Protocol for metadata and image collection at diabetic foot ulcer clinics: enabling research in wound analytics and deep learning. BioMed Eng OnLine 23, 12 (2024). https://doi.org/10.1186/s12938-024-01210-6), where a multimodal and comprehensive DFU data collection was conducted. While 2D imaging is the most accessible and feasible approach in DFU image collection, they significantly lack the essential contextual information required for healing assessment. Exploring or adding temperature factors (thermal imaging or just simple temperature values) may strengthen the scalability and practicality of the protocol, as this parameter is the most crucial element missing from the 2D DFU images.

The refence cited above has now been considered and included in the manuscript.

References:

Goldstein, H., 2011. *Multilevel statistical models*. John Wiley & Sons.

---

**VERSION 2 - REVIEW**

---

| | |
|---|---|
| **Reviewer** | **2** |
| **Name** | **Basiri, Reza** |
| **Affiliation** | **University of Toronto** |
| **Date** | **15-Nov-2024** |
| **COI** | |

---

The Author has addressed my concerns in the revision. I have no further comments.
